# Oncofertility care and influencing factors among cancer patients of reproductive age from Saudi Arabia

**DOI:** 10.3389/frph.2022.1014868

**Published:** 2022-11-17

**Authors:** Atlal Abusanad, Aseel Mohamed A. Mokhtar, Saad Adel A. Aljehani, Khaild Fuad A. Aljuhani, Khalid Abdullah A. Saleh, Baraa Hameed Alsubhi, Raad Mohammed Hamdi, Ammar Dawood Alzoriri

**Affiliations:** ^1^ Medical Oncology, King Abdul Aziz University Hospital, Jeddah, Makkah, Saudi Arabia; ^2^ Faculty of Medicine, King Abdulaziz University, Jeddah, Makkah, Saudi Arabia

**Keywords:** oncofertility, fertility, cancer, fertility preservation, competency framework, gonadotoxicity, chemothearpy, decision making

## Abstract

**Background:**

More cancer survivors and a greater burden of long-term side effects have resulted from rising cancer incidence, improved treatment modalities, and younger age at cancer diagnosis. Treatment- related Infertility (TRI) is a well-known sequelae. This study looked at current oncofertility support and fertility preservation (FP) in men and women of reproductive age with cancer in Saudi Arabia, where there is little knowledge on the subject.

**Methods:**

A cross-sectional study included oncology patients of reproductive age from an academic hospital was conducted. Patients' characteristics, cancer type, treatment modalities and assessment of oncofertility support data were collected and examined to assess oncofertility support and potentially influencing factors.

**Results:**

Our study included 135 patients (39.3% males and 60.7% females). Although 66.7% believed they were fertile at the time of diagnosis, and more than half planned to have children in the future, Unfortunately, only 37.8% have received fertility counseling, and only 17% have seen a fertility specialist. In male patients, the most common FP method was sperm cryopreservation (6.7%), while the majority of both genders (87.4%) did not use any FP method. Two-thirds of the patients are knowledgeable about TRI and FP methods. About half of the female patients (57.3%) were advised about the possibility of post-treatment amenorrhea while only 8.1% of the whole cohort received psychological support.

**Conclusions:**

Despite patients' satisfactory knowledge of TRI and FP, oncologists infrequently referred their patients to a specialized fertility service. More than half of our patients expressed a desire to have children in the future, but this desire was impeded by limited oncofertility care and FP procedures. Several factors influenced the knowledge of TRI, fertility counseling and FP. It is critical to incorporate oncofertility into management planning as it has a significant impact on patients' quality of life.

## Introduction

Incidence of different cancers is increasing globally and locally. According to WHO, there are more than 25,000 new cases annually in Saudi Arabia in the past decade, with more than 80,000 prevalent cases (1). Seventy percent of the Saudi population is younger than 40 years, with a life expectancy that is expected to increase to 78.4 (males) and 81.3 (females) by 2,050 and increasing population capacity coupled with the projected increase in cancer incidence by 60% in 2,030 compared to 2015. In addition to significant improvement in cancer treatments, all these factors are leading to better survival and more cancer survivors, specifically among the reproductive age patients. On the other hand, several cancer treatments have been linked to potentially long-term side effects, one of which is infertility. Overall, this suggests that TRI is an emerging issue that should be addressed among cancer patients (2).

Various chemotherapeutic agents have gonadotoxic effects with multiple pathophysiological mechanisms that are not fully understood. Basic cellular processes may be disrupted, and cell growth may be hampered, as a result of the gonadotoxic effect (3). The most vulnerable cells to the toxic effect of chemotherapy are those with high replication rate such as reproductive organs. The alkylating agents interfere with deoxyribonucleic acid (DNA) strands replication during cleavage by a covalent bond created between the strands (3, 4). Cancer treatment temporarily or permanently affects the fertility potential of between 50% and 75% of cancer survivors (5). It is associated with poor quality of life, low self-esteem, changes to body image, and psychological distress necessitating appropriate fertility-related psychological support, moreover, infertility can cause fear of abandonment or refusal from the partner and may affect sexual intercourse by making the conception as the main goal, the sexual pleasure and spontaneity are lost (6).

Oncofertility support is still a new topic in our region, with many religious and cultural factors influencing its application and feasibility (7). Few studies on this crucial topic had previously been conducted in any Arab country. According to a survey of oncologists from Saudi Arabia, while the majority of respondents (86.4%) did not refer their cancer patients to a fertility specialist, 90% of them believed patients would have benefited from fertility counseling, indicating suboptimal practice (8). These findings are similar to those of another study conducted in the United States, which found that, while the total number of patients receiving FP counseling has increased over time, only 9.8% of all fertile patients with new cancer diagnoses were referred for fertility counseling in 2011. Likewise, S. Logan and colleagues found that 62% of patients had unmet FP needs (9). Prior Saudi research focused on oncologists rather than patients. Although learning how local oncologists practice is important, it may not reflect the actual care that patients receive. Medical professionals should discuss the topic of infertility with cancer patients who are in their reproductive years or with the parents or legal guardians of children as soon as possible. To assist doctors with advice on fertility preservation, guidelines are periodically updated. Although there was an international guideline that was updated in 2018 by American Society of Clinical Oncologists (ASCO), there was no local guidelines that addressed local aspects (10).

The purpose of this study is to assess the oncofertility support that is offered to cancer patients of reproductive age, identify potentially influencing factors, investigate the current oncologists’ practice regarding fertility preservation and to measure the layman knowledge of FP among cancer patients. It is essential to have a better understanding of local oncofertility services provided by treating team and referral patterns for fertility support and related factors to ensure that patients' needs are met throughout the course of cancer treatment and survivorship and to implement pathways and design guidelines considering local barriers and practice (11, 12).

## Materials and methods

### Study design, setting, and participants

This is a descriptive cross-sectional study conducted at King Abdulaziz University Hospital, Jeddah, Saudi Arabia. All cancer patients in the daycare unit were included, and those who were previously known to be infertile or above age of 45 were excluded.

### Data collection

The authors designed a structured survey to collect data on awareness, knowledge, and behavior related to oncofertility support. Data was collected over a five-month period, from August 30, 2021 to January 29, 2022. The questionnaire is divided into four parts: The first section obtains consent, while the second section collects social and demographic data including: age, religion, gender, number of living children, marital status at diagnosis, region, level of education, family income, and whether or not the participant worked in the medical field. The third section used 14 single-response multiple-choice items to assess awareness, knowledge, and behavior of oncofertility support, while the fourth section assessed partner or guardian knowledge and behavior. The questionnaire and scoring methods are presented in (Supplementary Material 1).

### Procedure for data collection

Following ethical approval, a Google Forms questionnaire was created in order to interview cancer patients by asking them the questions on the questionnaire to ease the process.

### Ethical consideration

Ethical approval was obtained from the Institutional Review Board (IRB) at King Abdulaziz University Hospital, Jeddah, Saudi Arabia, in accordance with the Declaration of Helsinki. (Ref. No. 407-21). All data were kept confidential and were available only to the research team.

### Statistical analysis

Statistical analysis performed using SPSS software (version 23). Data were presented as means and standard deviations (SD) for continuous variables, we assumed normal distribution, while frequencies and percentages for categorical variables to determine the difference in knowledge scores between the two groups. The Chi-square test of independence was applied to determine the association between categorical variables including socio-demographic factors, knowledge scores, and fertility counseling score. fertility counseling score. The significance level was considered as less than or equal to 0.05.

Statistical analysis performed using Statistical Package for Social Sciences (SPSS) software (version 23).

## Results

**Figure 1:**
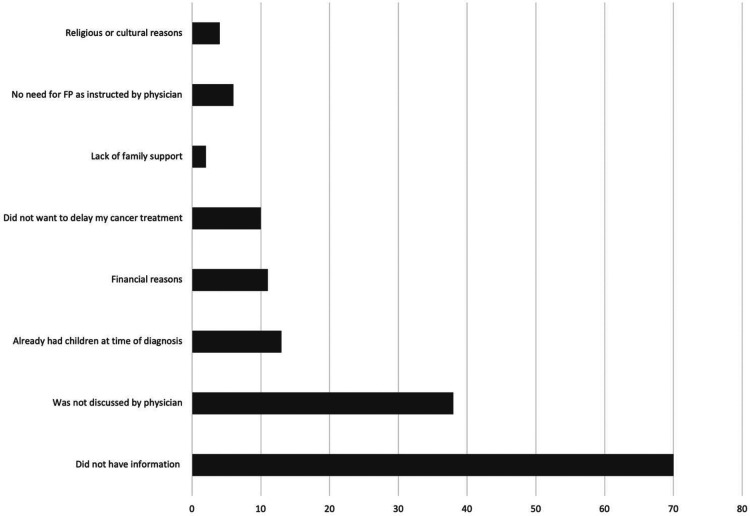
Reasons for not undergoing fertility preservation.

**Figure 2:**
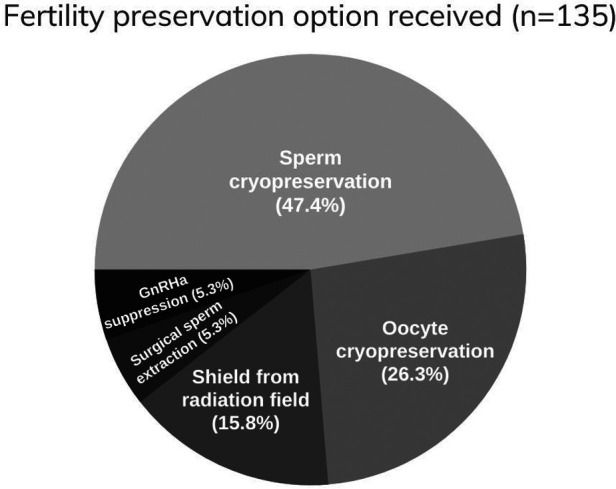
Interventions of fertility preservation offered to patients.

Female patients outnumbered their male counterpart. The majority were between the ages of 30–45 years old. On the assumption that the maximum age for being fertile is 45, this age was set as the upper limit for including patients in the survey. Most of the patients received an education that at the level of high school or higher and only 8.2% of patients have income over 15,000 SAR (3999 USD) per month (Table 1). More than half of the participants expressed the desire to have children in the future and nearly two-thirds believed that they are fertile at the time of diagnosis (Table 2). Breast cancer was the most common, followed by bladder, testicular cancers and leukemia (Table 3). About half of the female patients (57.3%) were advised about the possibility of post-treatment amenorrhea while only 8.1% of the whole cohort received psychological support. Treatment modalities are listed in (Table 4). Two third of the patients (70.4%) have a satisfactory knowledge of TRI and FP methods, while 29.6% have poor knowledge. Males are more knowledgeable than females with satisfactory knowledge of 75.5%, and 67.1%, respectively. Patients with higher education and income were more likely to demonstrate a higher knowledge score (Table 5). Almost two-thirds (62.2%) of patients were not offered fertility counseling by their physicians. Gender influenced physicians' decision to offer fertility counseling, as our data shows a significant difference in the proportion of male and female patients who received counseling (49.1% and 30.5% respectively) (*P*-value = 0.030). Likewise, patients with higher income were more likely to receive fertility counseling. Age, educational level and family size did not impact fertility counseling assessment (Table 6). Obstacles to FP are illustrated in (Figure 1) and different FP methods which were received by the participants are shown in (Figure 2).

**Table 1:** Sociodemographic characteristics of patients.

		Frequency	Percent
Gender	Male	53	39.26%
	Female	82	60.74%
Age groups	Age less than 16	12	8.89%
	17–30	29	21.48%
	31–45	94	69.63%
Education level	Less than high school	25	18.52%
	High school	43	31.85%
	Bachelor	51	37.78%
	Diploma	16	11.85%
Income of the family (Per month)	Less than 5,000 SAR (1333 USD)	60	44.44%
	5,000–10,000 SAR (1333-2666 USD)	42	31.11%
	10,000–15,000 SAR (2666-3999 USD)	22	16.30%
	More than 15,000 SAR (3999 USD)	11	8.15%
Family size (number of living children)	0	46	34.07%
	1	8	5.93%
	2	26	19.26%
	3	22	16.30%
	4	19	14.07%
	5	7	5.19%
	6	7	5.19%
Marital status at the time of diagnosis	Married	84	62.22%
	Single	42	31.11%
	Divorced	8	5.93%
	Widow	1	0.74%
Total		135	100

**Table 2:** The perception of being fertile and the desire to have children in the future.

		Frequency	Percent
Fertility status at time of diagnosis	Fertile	90	66.67%
	Non-fertile	4	2.96%
	I don't know	41	30.37%
Intention to have children in future	Yes	76	56.30%
	No	26	19.26%
	Maybe/not yet decided	33	24.44%
Were you pregnant at the time of diagnosis (*n* = 82)	Yes	3	3.66%
	No	79	96.34%

**Table 3:** Types of cancer.

Breast cancer	48 (35.56%)
Bladder cancer	24 (17.78%)
Testicular cancer	18 (13.33%)
Leukemia	11 (8.15%)
Ovarian cancer	9 (6.67%)
Colorectal cancer	7 (5.19%)
Kidney cancer	6 (4.44%)
Other cancers	12 (8.89%)

**Table 4:** Management plan and treatment modalities.

Patients received psychological help after diagnosis		11 (8.1%)
Female patients informed of the possibility of amenorrhea (*n* = 82)		47 (57.3%)
Treatment modality	Radiotherapy	44 (32.6%) (*n* = 135)
	Chemotherapy	111 (82.2%) (*n* = 135)
	Hormonal therapy	25 (18.51%) (*n* = 135)
	Surgical therapy	111 (82.2%) (*n* = 135)

**Table 5:** Assessment of TRI and FP knowledge/awareness among cancer patients.

Gender + total		Male	Female	Total frequency	Percent
Knowledge assessment score	Poor knowledge	13 (24.5%)	27 (32.9%)	40	29.63%
	Satisfactory knowledge	40 (75.5%)	55 (67.1%)	95	70.37%
*P* value = 0.297 (not significant)					
Age		Less than 16		17–30	31–45
Knowledge assessment score	Poor knowledge	6 (50%)		8 (27.6%)	26 (27.7%)
	Satisfactory knowledge	6 (50%)		21 (72.4%)	68 (72.3%)
*P* value = 0.270 (not significant)					
Education level		Diploma	Less than high school	High school	Bachelor
Knowledge assessment score	Poor knowledge	4 (25%)	17 (68%)	14 (32.6%)	5 (9.8%)
	Satisfactory knowledge	12 (75%)	8 (32%)	29 (67.4%)	46 (90.2%)
*P* value = 0.000 (significant)					
Income of the family		Less than 5,000 SAR	5,000–10,000 SAR	10,000–15,000 SAR	More than 15,000 SAR
		(1333 USD)	(1333–2666 USD)	(2666–3999 USD)	(3999 USD)
Knowledge assessment score	Poor knowledge	24 (40%)	13 (31%)	2 (9.1%)	1 (9.1%)
	Satisfactory knowledge	36 (60%)	29 (69%)	20 (90.9%)	10 (90.9%)

*P* value = 0.020 (significant).

Poor knowledge = 0–1, Satisfactory knowledge = 2–3, from 3 questions [Do you have any idea that cancer will affect your future fertility? Are you aware of the effect of chemotherapy/radiotherapy on future fertility and ovarian function? Are you aware of available Fertility Preservation (FP) options for you?] each question if answered yes will get 1 point. TRI = treatment-related infertility, FP = fertility preservation.

**Table 6:** Assessment of the fertility counseling provided by physicians.

Gender + total		Male		Female	Total
Fertility counseling assessment	Lack of fertility counseling	27 (50.9%)	57 (69.5%)	84 (62.2%)	
	Fertility counseling offered	26 (49.1%)	25 (30.5%)	51 (37.8%)	
*P* value = 0.030 (significant)					
Age		Less than 16		17–30	31–45
Fertility counseling assessment	Lack of fertility counseling	8 (66.7%)		17 (58.6%)	59 (62.8%)
	Fertility counseling offered	4 (33.3%)		12 (41.4%)	35 (37.2%)
*P* value = 0.873 (not significant)					
Education level		Less than high school	High school	Diploma	Bachelor
Fertility counseling assessment	Lack of fertility counseling	20 (80%)	25 (58.1%)	12 (75%)	27 (52.9%)
	Fertility counseling offered	5 (20%)	18 (41.9%)	4 (25%)	24 (47.1%)
*P* value = 0.084 (not significant)					
Family size		0		1 or 2	3 or more
Fertility counseling assessment	Lack of fertility counseling	29 (63%)		22 (64.7%)	33 (60%)
	Fertility counseling offered	17 (37%)		12 (35.3%)	22 (40%)
*P* value = 0.084 (not significant)					
Income of the family		Less than 5,000 SAR	5,000–10,000 SAR	10,000–15,000 SAR	More than 15,000 SAR
		(1333 USD)	(1333–2666 USD)	(2666–3999 USD)	(3999 USD)
Fertility counseling assessment	Lack of fertility counseling	48 (80%)	23 (54.8%)	9 (40.9%)	4 (36.4%)
	Fertility counseling offered	12 (20%)	19 (45.2%)	13 (59.1%)	7 (63.6%)

*P* value = 0.001 (significant).

Lack of fertility counseling = 0, Fertility counseling offered = 1–2, from 2 questions (Did the doctor discuss fertility-related options with you before starting your therapy? Have you ever been seen by a fertility specialist before initiating treatment?) each question if answered yes will get 1 point.

## Discussion

Although more than half of the participants expressed the desire to have children in the future and nearly two-thirds believed that they are fertile at the time of diagnosis, only 37.8% of our sample received fertility counseling and 14% underwent actual FP technique. According to a study conducted in Riyadh, about 76% of oncologists believe that providing fertility support is crucial, and 90% believe that patients would benefit from being referred to a fertility specialist for counseling. However, the majority (86.4%) of doctors did not refer their cancer patients (13), which is mirrored in our study where 83% of the patients were not referred to a fertility specialist before receiving treatment. Less than a quarter of oncologists reported referring patients to reproductive specialists for FP, according to a research from the United States. This is contrary to the American Society of Clinical Oncology (ASCO) recommendations that states health care providers should counsel their cancer patients regarding potential infertility prior to initiating any treatment (13, 14). We observed that the most frequently reported reason for patients not receiving FP is insufficient information, followed by lack of discussion by physicians and having children at the time of diagnosis (Figure 1). Some studies showed that the majority of the physicians reported that they never received courses or training regarding FP (15). A low percentage of referrals could be multifactorial. Hence, lack of awareness among oncologists and gynecologists, lack of advancements in early cancer diagnosis and treatment, low referrals from oncologists, poor inter-institutional communication, and the absence of oncofertility specialists are recognized medical impediments (16). This research will guide the effective implementation of solutions by identifying underlying limitations of oncofertility application locally.

Higher education and income were associated with better knowledge of the potential effects of treatment on fertility and FP methods among our patients, likewise patients with higher income were more likely to receive fertility counseling than the ones with lower income. This finding can be attributed to the lack of health insurance coverage for fertility services, a lack of institutional and research support, and very high costs are all challenges to overcome; many fertility services are offered in private clinics and are paid for out-of-pocket (8, 11). The average cost of a single cycle of *in vitro* fertilization and intracytoplasmic sperm injection is widely variable, starting from 1,500 USD and reaching 10,000 USD. The barriers and challenges to oncofertility care in middle income countries were also similar to those in high-income countries with no service availability, high costs of FP methods and fears of delaying treatment (17, 18). Our patients identified financial reason among others for not receiving FP (Figure 1). Conservative religious, cultural, and ethical beliefs that prevent third-party reproduction in various nations are examples of social and legal hurdles (15), however, only small number of our patients recognized this as an obstacle for obtaining FP.

Gender significantly influenced physicians’ decision to offer fertility counseling, as our data showed a larger proportion of male patients received counseling than female patients. Additionally, the most reported FP technique was sperm banking among the whole cohort. Oocyte cryopreservation was infrequently reported. This was demonstrated despite the fact that female participants outnumbered their male counterpart and breast cancer accounted for the most common malignancy in this cohort indicating gender disparity which has to be addressed in future research. Men in this cohort were more knowledgeable about TRI and FP than women, which could have resulted in a self-promoting attitude and influencing their physicians to discuss fertility issues, as well. Furthermore, testicular cancer in young men will unavoidably raise the issue of fertility affection because it affects the organ of reproduction. Testicular cancer was the third commonest malignancy in our patients.

Breast cancer was the most frequently reported, followed by bladder, testicular cancers and leukemia. Our target population was cancer patients whose ages were below 45 years, this targeted age had an influence on the type of cancers in our study, high prevalent cancer like colorectal, prostate, and lung cancers were less detected in our survey due to the methodology that we used.

Psychological support is an important part of management plan for cancer patients and has been reported to reduce fertility-related psychological distress like fear of rejection, which may lead to an increase in the rate of divorce (7). Unfortunately, only 8.1% of our patients have received psychological support. In most cases, cancer treatment can't be delayed; a rapid referral for proper counseling must be considered.

Overall there is a need to address TRI and FP in young cancer patients routinely. Such discussion is essential to integrate within the treatment planning. Oncologists are encouraged to initiate discussion, assess patient needs, recommend suitable intervention and refer to a specialized fertility service whenever available. Oncofertility is a developing field in which significant progress has been made specifically for cancer survivors of reproductive age. Furthermore, cancer patients and survivors must be educated about this issue, which is reflected on the life's quality.

### Limitations of the study

The questionnaire was administered electronically, which may have prompted self-disclosure, and it is influenced by psychological and environmental factors during the response to the questions. Lack of information about the type of chemotherapy and radiotherapy given to patients would limit our understanding of the severity of gonadotoxicity, which varies depending on the chemotherapeutic agent used and the location of radiotherapy.

## Conclusion

Although patients showed satisfactory knowledge of TRI and FP, oncologists infrequently referred their patients to a specialized fertility service. More than half of our patients expressed a desire to have children in the future, however this desire was faced with limited oncofertility care and a few fertility preservation procedures. Patients with higher income had a higher likelihood of having oncofertility care. Fertility counseling and care among cancer patients must be addressed with equity and equality regardless of any specific personal or societal considerations. It must be incorporated into management planning to enhance post-treatment survivorship and life's quality.

## Data Availability

The raw data supporting the conclusions of this article will be made available by the authors, without undue reservation.
