# Supplementary material for: Oncofertility care and influencing factors among cancer patients of reproductive age from Saudi Arabia
Source: Front Reprod Health. 2022 Nov 17;4:1014868. doi: 10.3389/frph.2022.1014868 (PMC9714457; doi:10.3389/frph.2022.1014868)
Supplement: Supplementary file 1 [file Datasheet1.pdf]

Questionnaire: oncofertility support among cancer patients ENGLISH version:

Preferred language

- English
- عربي

CONSENT:

This study aims to assess the clinical provision of preserving fertility support among specific populations in Saudi Arabia. All the responses you provide for this study will remain confidential. When the study results are reported, you will not be identified by name or any other information that could be used to infer your identity. Thank you for your time.

- Agree

Assessment of oncofertility support clinical provision:

MRN.

- ()

Age:

- ()

Religion:

- Muslim
- Non-muslim

Gender:

- Female
- Male

Family size (number of living children):

- ()

Marital status at the time of diagnosis:

- Married
- Single

- Divorced
- Widow

Region:
- Western
- Southern
- Eastern
- Northern
- Middle

Education:
- Less than high school
- High school
- Diploma
- Bachelor
- Higher degree

Income of the family:
- Less than 5000
- 5000 - 10000
- 10000 - 15000
- More than 15000

Profession:
- Health-related
- Non-health-related

Have you ever been seen by a fertility specialist before initiating treatment?
- Yes, referred by doctor
- Yes, by myself

Fertility status at the time of diagnosis:
- Fertile
- Not fertile
- I don’t know

Type of cancer:
- Skin cancer.
- Lung cancer.
- Prostate cancer.

- Breast cancer.
- Colorectal cancer.
- Kidney (renal) cancer.
- Bladder cancer.
- Lymphoma.
- Leukemia
- Sarcoma
- Other

What was your age at the time of your cancer diagnosis?

- ()

Do you intend to have children?

- Yes
- No
- Maybe / not yet decided

Do you have any idea that cancer will affect your future fertility?

- Yes
- No

Are you aware of the effect of chemotherapy/radiotherapy on future fertility and ovarian function?

- Yes
- No

Of these, what type of treatment have you received / are planning to receive for cancer?

- Surgery
- Chemotherapy
- Radiotherapy
- Hormonal

Are you aware of available Fertility Preservation (FP) options for you?

- Yes
- No

If you answered 'Yes' in the previous question please choose all methods that you know from the following:

- Oocyte cryopreservation

- Embryo cryopreservation
- Ovarian cortex preservation
- Shield from radiation field
- GnRHa suppression
- Ovarian transposition
- Sperm cryopreservation
- Surgical sperm extraction
- Immature testicular tissue cryopreservation
- Other

Did the doctor discuss fertility-related options with you before starting your therapy?
- Yes
- No
- Maybe

What is/was the reason for not using Fertility Preservation (FP) options before treatment?
- Did not have information
- Financial reason
- Lack of family support
- Was not informed by physician
- Did not want to delay my cancer treatment
- Risk of transmission to the future child children
- Already had desired at that time
- Religious / cultural causes
- Other

Did you receive any psychological help after you have been diagnosed?
- Yes
- No
- Maybe

Did you receive any of the following fertility preservation options?
- Oocyte cryopreservation
- Embryo cryopreservation
- Ovarian cortex preservation
- Shield from radiation field
- GnRHa suppression
- Ovarian transposition
- Sperm cryopreservation

- Surgical sperm extraction
- Immature testicular tissue cryopreservation
- Didn't use any method

FOR MALE:

Do you have a partner or guardian?

- I have partner
- I have guardian
- No

FOR FEMALE:

Do you have a partner or guardian?

- I have partner
- I have guardian
- No

Did the doctor discuss the possibility of amenorrhea with you before starting your therapy?

- Yes
- No

Were you pregnant at the time of diagnosis?

- Yes
- No

Questions for your partner:

Partner age:

- ()

Did the partner discuss fertility-related options with you?

- Yes
- No

Is fertility an important issue for you as a partner?
- Yes
- No
- Maybe

Did you notice any difference in the sexual activities after the diagnosis?
- More / increased
- Less / decreased
- No change

Partner education level:
- Less than high school
- High school
- Diploma
- Bachelor
- Higher degree

For male partner, the number of current wifes?
- 1
- 2
- 3
- 4
- The partner is female

Questions for the guardian:

Age of the guardian?
- ()

Relativity of the guardian?
- Father
- Mother
- Brother
- Sister
- Other

Did the doctor discuss fertility-related options with you as the guardian?

- Yes
- No

Guardian education level?

- Less than high school
- High school
- Diploma
- Bachelor
- Higher degree

نسخة باللغة العربية

اللغة المفضلة

- العربية
- English

الموافقة على المشاركة

تهدف هذه الدراسة إلى تقييم الخدمات الطبية المقدمة في مجال الأورام والحفاظ على الخصوبة في المملكة العربية السعودية، ستبقى جميع الردود التي تقدمها لهذه الدراسة سرية. عند استخراج نتائج الدراسة، لن يتم تحديدك بالاسم او بأي معلومات أخرى يمكن استخدامها لاستنتاج هويتك. شاكرين لك وقتك الثمين

- أوافق على المشاركة

تقييم الخدمات الطبية المقدمة في مجال الأورام والحفاظ على الخصوبة

رقم ملف المريض

* العمر

* الديانة

-مسلم

-غير مسلم

* الجنس

-ذكر

-أنثى

* عدد الأبناء الأحياء

* الحالة الاجتماعية عند التشخيص

-متزوج/ة

-أعزب/ عزباء

مطلق/ة-

-أرمل/ة

المنطقة

الغربية-

-الجنوبية

-الشرقية

الشمالية-

-الوسطى

* المستوى التعليمي

-أقل من الثانوية

-الثانوية

-دبلوم

-بكالوريوس

-دراسات عليا

* الدخل العائلي

-أقل من 5000

-من 5000-10000

-من 10000-15000

-أكثر من 15000

* المجال الوظيفي

-في المجال الصحي

-خارج المجال الصحي

-غير موظف

* هل سبق وان تم تقييمك من قبل طبيب متخصص بالخصوبة

-نعم، تم تحويلي من قبل الطبيب

-نعم، ذهبت بنفسي

-لا

* القدرة على الإنجاب عند التشخيص

-قادر/ة على الإنجاب

-غير قادر/ة على الإنجاب

-لا أعلم

* نوع السرطان

في حال تشخيصك بنوع اخر الرجاء كتابته

-سرطان الجلد

-سرطان الرئة

-سرطان البروستات

-سرطان الثدي

-سرطان القولون / المستقيم

-سرطان الكلى

-سرطان المثانة

-سرطان الغدد اللمفاوية

-سرطان الدم

سرطان الساركوما-

-أخرى

* عمرك عند تشخيصك بالسرطان

* مستقبلا هل لديك الرغبة بالإنجاب

نعم-

-لا

ربما / لم أقرر بعد-

* خصوبتك مستقبلا هل لديك فكرة بأن السرطان قد يؤثر على

-نعم

-لا

* ؟هل أنت على دراية بالتأثير الضار للعلاج الكيميائي\الإشعاعي على الغدد التناسلية ووظيفة المبيض والخصوبة في المستقبل

-نعم

-لا

* ؟ما نوع العلاج الذي تلقيته\تخطط لتلقيه لعلاج السرطان

في حال استعمال أكثر من خيار طبي الرجاء اختيارها جميعها

الجراحة-

العلاج الكيميائي-

-العلاج الإشعاعي

العلاج الهرموني-

* ؟هل أنت على دراية بخيارات حفظ الخصوبة الممكنة لك

-نعم

-لا

اذا جاوبت بـ"نعم" في السؤال السابق، اختر من التالي الطرق التي تعرفها

-تجميد البويضات

-تجميد الأجنة

حفظ جزء من المبيض-

-واقي مضاد للأشعة العلاجية

-مثبطات الهرمون المطلق لهرمونات الغدد التناسلية

تبديل المبيض-

حفظ وتجميد الحيوانات المنوية-

استخراج الحيوانات المنوية جراحيا-

-حفظ انسجة الخصية غير مكتملة النمو

:أخرى

* ؟هل ناقشك طبيبك المختص عن خيارات حفظ الخصوبة قبل بدئك للعلاج

نعم-

-لا

-ربما

* السبب في عدم تلقيك احد خيارات حفظ الخصوبة قبل العلاج ما هو

-سبق و ان تلقيت احد هذي الاجراءات

ليس لدي اي معلومات بهذا الخصوص-

-اسباب مالية

-الافتقار للدعم العائلي

لم يتم اعلامي من قبل الطبيب المختص-

-لم ارغب في تأخير العلاج

-الخوف من توارث المرض للذرية

-لدي ابناء كفاية ولا ارغب بالمزيد

-اسباب دينية / مجتمعية

:أخرى

* هل تلقيت اي دعم نفسي من متخصص بعد تشخيصك

-نعم

-لا

-ربما

* :خيارات حفظ الخصوبة التالية هل سبق وان تلقيت أحد

-تجميد البويضات

-تجميد الاجنة

-حفظ جزء من المبيض

واقي مضاد للأشعة العلاجية-

-مثبطات الهرمون المطلق لهرمونات الغدد التناسلية

-تبديل المبيض

-حفظ وتجميد الحيوانات المنوية

استخراج الحيوانات المنوية جراحية-

-حفظ انسجة الخصية غير مكتملة النمو

-لم استخدم أي طريقة من قبل

:أخرى

للذكور

* هل لديك زوجة أو ولي أمر

نعم، لدي زوجة-

-نعم، لدي ولي أمر

-لا

للإناث

* هل لديك زوج او ولي امر

-نعم، لدي زوج

-نعم، لدي ولي أمر

-لا

* هل سبق أن ناقشك طبيبك عن احتمالية انقطاع الدورة الشهرية بعد العلاج

-نعم

-لا

* هل كنت حامل عند تشخيصك بالسرطان

-نعم

-لا

أسئلة للزوج

* العمر

* ؟هل تم النقاش فيما بينكما عن الخصوبة وخيارات حفظ الخصوبة

-نعم

-لا

* ؟هل الخصوبة من المواضيع الهامة لدى شريكك الزوجي

-نعم

-لا

-ربما

* ؟هل هناك اختلاف أو فرق في العلاقة الجنسية بعد التشخيص والعلاج عما قبلهما

-زادت

-نقصت

-لم تتغير

* المستوى التعليمي للزوج

-اقل من الثانوي

-الثانوية العامة

-دبلوم

-بكالوريوس

-دراسات عليا

للذكور، عدد زوجاتك الحالي

1-

2-

3-

4-

أسئلة لولي الامر

* العمر

* صلة القرابة

-أب

-أم

أخ-

أخت-

أخرى:

* هل ناقشك الطبيب المختص بخيارات حفظ الخصوبة بصفتك ولي الامر قبل بدأ العلاج

-نعم

-لا

* المستوى التعليمي لولي الامر

اقل من الثانوي-

الثانوية العامة-

-دبلوم

-بكالوريوس

-دراسات عليا

Analysis objectives/ questionnaire scoring

1- descriptive analysis for the patients characteristics and the study variables

2- assess the knowledge/ awareness of cancer patients re: fertility preservation. Therefore, I suggest that the question evaluating the knowledge/ awareness are grouped and answers are given points to create a scale as follows:

	yes	no
Do you have any idea that cancer will affect your future fertility?	1	0
Are you aware of the effect of chemotherapy/radiotherapy on future fertility and ovarian function?	1	0
Are you aware of available Fertility Preservation (FP) options for you?	1	0

Poor knowledge/awareness = 0-1
Satisfactory knowledge/awareness = 2-3

3- assess the fertility counseling for cancer patients. Therefore, I suggest that the question evaluating the fertility counseling are grouped and answers are given points to create a scale as follows:

Did the doctor discuss fertility-related options with you before starting your therapy?	1 if answer was yes or maybe	0 if answer was no
Have you ever been seen by a fertility specialist before initiating treatment?	1 if answer was referred by the doctor	0 if answer was no or referred by myself

0 = lack of fertility counseling
1-2 = fertility counseling offered

4- examine the association between the level of knowledge/awareness of fertility preservation among cancer patients and different factors as follows:

factors	Poor awareness	Satisfactory awareness	p- value
Age at cancer diagnosis			
Gender			
Family size			
Married vs. unmarried			
Education			
income			
profession			
Desire to have children (yes or unsure vs. No)			
Type of cancer			
Age at cancer diagnosis			

5- assess the association between the offering of fertility counseling and different factors

factors	Lack of counseling	Offered counseling	p- value
Age at cancer diagnosis			
gender			
Married vs. unmarried			
Family size			
Desire to have future children			
Type of treatment			
Type of cancer			
Psychosocial help			

6- assess additional contributing factors that influencing fertility knowledge/awareness among cancer patient with partners (the analysed sample is going to be the ones with partners).

factors	Poor awareness	Satisfactory awareness	p-value
Partner age			
Partner education level			

Discussion of fertility with the partner			
The importance of fertility to the partner			
Number of wives if male partner			

7- descriptive analysis of

- the most frequent barrier to fertility preservation,
- most common type of fertility preservation if used
- sexual behaviour after diagnosis for the one with partner
- pregnancy rate at diagnosis if answered yes
- frequency of post-treatment amenorrhea discussion in female patients
